# The unexplored territory of aesthetic needs and the development of the Aesthetic Needs Scale

**DOI:** 10.1371/journal.pone.0299326

**Published:** 2024-03-18

**Authors:** Agata Hiacynta Świątek, Małgorzata Szcześniak, Hanna Borkowska, Michał Stempień, Karolina Wojtkowiak, Rhett Diessner

**Affiliations:** 1 Instytut Psychologii, Uniwersytet Szczeciński, Szczecin, Poland; 2 Psychology Department, Lewis-Clark State College, Lewiston, Maine, United States of America; 3 Bahá’í Institute of Higher Education, Tehran, Iran; Novi Sad School of Business, SERBIA

## Abstract

Human needs, and their fulfillment, are the building blocks of human development, personality, and well-being. However, no published paper in the field of psychology has focused on exploring *aesthetic needs*. Maslow (1986) gave the topic little more than a paragraph; and Dweck [1], in her elegant Unified Theory of Motivation, Personality, and Development, never mentions aesthetic needs. The aim of this article is to describe developing a scale for measuring the intensity of aesthetic needs. The structure, psychometric properties, and criterion-related validity of the scale were verified with three independent samples (total *N* = 592). The results of an EFA and two CFAs indicated a three-factor structure: 1) the need to aestheticize everyday life (aesthetic experiences of everyday objects and events unrelated to art, such as the presentation of food or the appearance of a workspace, etc.); 2) the need for contact with aesthetic creations (the arts); 3) the need to aestheticize the built and natural environments (urban spaces, architecture, parks, wild nature, etc.). In addition, our criterion-related convergent validity studies have shown that people with high aesthetic needs are characterized by experiencing more intense experiences in contact with works of art, have higher aesthetic competence in art, are more intensely involved in four forms of beauty, have a higher ability to integrate beauty, a stronger trait gratitude, curiosity about nature, greater sensitivity to disgust, and the need for internal and external stimulation. This scale may prove useful in research on individual differences and the psychology of aesthetics.

## Introduction

The importance and role of *needs* in human development and flourishing cannot be over-emphasized. In Dweck’s Unified Theory of Motivation, Personality, and Development, needs are the central building blocks of the human journey. Needs are the foundation of what humans seek in life, they are the basis of their personality traits, and create the conditions for lifelong development. Dweck states, “the key criteria for a need, basic or otherwise, are (a) that there is chronic, high, and universal value attached to the goals that serve it and (b) that successfully attaining goals related to that need is important for optimal well-being in the present and optimal psychological development in the future” [1, p. 690].

Maslow [2] initially posited five somewhat hierarchical levels of basic need, but later in his life he revised that to also include *aesthetic needs* (as well as cognitive and self-transcendence needs). Maslow wrote, “We know even less about these [aesthetic needs] than about the other [needs]… I have attempted to study this phenomenon on a clinical-personological basis… and… in some individuals there is a truly basic aesthetic need… they crave actively, and their cravings can be satisfied only by beauty… It is seen almost universally in healthy children” (p. 25). However, Maslow [2] devoted less than half of a page to explaining aesthetic needs in the entire book, and never offered any empirical studies on the topic. Likewise, in Rollo May’s book [3], *My Quest for Beauty*, he never once used the phrases “aesthetic needs” nor “the need for beauty.” And this is the same with Dweck [1]; she never mentions aesthetic needs or beauty in her paper on Unified Theory, despite Maslow assuring us that the craving (need) for beauty is found universally among developing children.

Entering the phrase “aesthetic needs” into PsycInfo yields 13 articles from academic journals, but 10 of those 13 studies only deal with aesthetic needs (AN) briefly, cursorily, and/or non-empirically. Of the other three papers, one is a thoughtful qualitative reflection about the importance of nurses fulfilling the aesthetic needs of patients [4]; the other two concern the development of a measure of quality of life in the workplace [5,6]. It appears there is no published scale to comprehensively measure aesthetic needs (AN). We intend to remedy that lacunae.

How important are aesthetic needs and the need for beauty? Despite the lack of theoretical and empirical work on aesthetic needs, they are a very important but empirically unexplored territory in the psychological literature. The Western canon is comprised by three great values, Truth, the Good, and Beauty [7]; and beyond the West, for example, these values were treasured by Confucius [8]. As Danto [9, pp. 14–15] has written, I “came to view that in writing about beauty as a philosopher, I was addressing the deepest kind of issue there is. Beauty is but one of an immense range of aesthetic qualities… But beauty is the only one of the aesthetic qualities that is also a virtue, like truth and goodness. It is not simply among the values we live by, but one of the values that defines what a fully human life means”. And further, May [3, p. 27], wrote, “When Plato considered the great trilogy of Beauty, Truth, and Goodness, he placed Beauty at the top because Beauty is harmony, and whether Truth or Goodness are harmonious is the test of their integrity”.

### Coherency, integration, and aesthetic needs

Dweck [1] posits three basic needs, which arise immediately or early in the first year of life: acceptance, predictability, and competence. She notes that next, later in the first year, the need for trust surfaces (a combination of acceptance and predictability); then in the second year the need for control/autonomy is expressed (a combination of predictability and competence); which is followed by the need for esteem (a twining of acceptance and competence). She particularly, however, highlights the importance of the need for self-coherence: “the need for self-coherence that sits at the intersection of all of the psychological needs and represents the need to feel psychologically rooted and intact” (p. 693). This need arises between the middle and end of the first year of life and integrates the three basic needs. Furthermore, she demonstrates that identity and meaning are critical sub-needs of the need for self-coherency. Although Dweck does not explicitly describe aesthetic needs, we believe they are a part of, or emerge from, the need for self-coherency. We think so, because aesthetics and beauty bring so much meaning to life [10] and aesthetic sensitivity is a key aspect of identity in general [11], and particularly moral identity and identifying with nature (Diessner et al., 2018b) [12].

Related to self-coherency is Ferrucci’s [13] concept of the ability to integrate beauty into our minds and being. Coherency can be defined as the ability to *integrate* the various needs, motivations, and aspects of selfhood. Ferrucci (2009) describes aesthetic intelligence as having three aspects, (a) intensity of beauty experiences, (b) range of beauty experiences, and the most important, (c) the ability to integrate beauty into our minds, emotions, and behavior. He suggests that contact with what a person perceives as beautiful, through internalization of the experience, allows for inner transformation (changing one’s thoughts about oneself, the world, and others). The extent to which this transformation is lasting is unknown, but we assume that contact with subjectively perceived beauty could be, or should be, an aspect of the process of self-actualization [14]. The ability to integrate beauty, achieved through goals arising from aesthetic needs, could serve the function of affirming self-coherency. This is reinforced in Kowalik’s work: "The artistic experience not only triggers a disruption in the stream of consciousness but also initiates the process of reordering our inner life" [15, p. 167].

### Three possible domains of aesthetic need

An aesthetic attitude, at least subjectively, determines what is *aesthetic* and what is not for each person [16]. Since nearly every element of reality has the potential to be perceived in an aesthetic manner [17], the question arises whether aesthetic needs are a homogeneous construct, a continuum of a general demand for beauty and other aesthetic qualities, or whether aesthetic needs are best divided into semi-discrete domains. Machniewicz [18] deliberated on the aesthetics of everyday life, assuming that every object can be seen as aesthetic, and every work of art is an aesthetic object, but not every aesthetic object is art. This suggests the existence of at least two categories of aesthetic needs: one related to everyday life (e.g., aesthetics of mundane objects) and the other related to works of art. It is known that not everyone is interested in art, nor finds it intriguing, nor wants to participate in it [19]. However, this does not necessarily mean that such individuals would be indifferent to their own clothing or the furnishings of the room in which they relax. In terms of everyday objects, the appearance of a product is considered a key factor in purchasing decisions [20]. There is a third possible category, the external appearance of the environment, such as buildings and landscapes, which also affects a person’s well-being and psychological states [21]. It also matters to homebuyers and developers. Environmental attributes, such as a view from a high floor, the presence of green areas, and proximity to bodies of water, lead to an increase in housing prices [22]. Thompson [23] postulated that appreciating the beauty of the natural environment and appreciating the beauty of works of art are similar. Studies on brain network activity provide evidence that a specific area of the cerebral cortex may be involved in perceiving various types of beauty [24,25]. Therefore, on the one hand, we can differentiate domains of aesthetics (such as, everyday aesthetics, artistic aesthetics, and aesthetics related to the built and natural environments), while, on the other hand, its perception may involve similar neural systems. Thus, various categories of aesthetic needs may demonstrate both domain generality and domain specificity, which is a common finding in brain research for various forms of beautiful stimuli [26].

It may be that many (or all?) people require aesthetic experiences related to the enjoyment of artistic and non-artistic forms of beauty, and other aesthetic stimuli, to develop greater levels of well-being, and to engage fully in self-actualization and self-transcendence [14]. Research on related constructs confirms the developmental potential of such aesthetic experiences. Engagement with beauty [27] and the ability to appreciate it contribute to individual and collective flourishing [12]. The character strength of appreciating beauty and excellence [28] is positively related to well-being and prosociality [29]. The ability to integrate experiences of beauty into our being, as one of the components of aesthetic intelligence [13], co-occurs with the Light Triad and various dimensions of spirituality [30]. Moreover, aesthetic competencies in art are positively related to positive emotions [31]; e.g., professional artists (painters) have demonstrated a higher level of happiness than non-artists [32]. It should be mentioned that aesthetic experiences do not necessarily have to be related to beauty, as Dietrich and Knieper [33] have discussed, but beauty remains an ultimate aesthetic value [34] and the prototypical aesthetic emotion [34].

As mentioned above, despite there being 13 hits in PsycInfo for “aesthetic needs,” there has been no substantial empirical study focused on addressing the *need* for contact with aesthetic stimuli (by "aesthetic stimuli," we mean any stimulus interpreted by an individual as aesthetic). In the context of art, there are studies that developed an aesthetic processing preferences scale [35] and also an aesthetic responsiveness scale [36]. However, both these scales focus on art, and not the broad range of possible aesthetic needs (which can include nature, interior décor, urban spaces, etc.).

There is also a scale that measures the *desire* for aesthetic experiences, across a broad range of aesthetic stimuli [37]; however, *desires* are not the same as *needs*. As Brock and Miller [38, n.p.] note, “[p]hilosophers who write about the concept of need are keen to emphasize how needs differ from desires, wants, and preferences: we often want things that we do not need, and equally we may not want what we need because we fail to recognize its importance to us.” Fulfilling psychological *needs* is essential to psychological health and human flourishing [1]; however, fulfilling desires may or may not be. Sometimes we desire things that we do not need; in fact, sometimes fulfilling desires damages our psychological health and prevents flourishing (drugs, sex with the wrong person, over-eating, etc.).

McManus and Furnham [19] analyzed the significance of various factors such as personality, education, interest in the frequency of aesthetic activities, i.e., art consumption. However, their work did not include the concept of aesthetic needs. Despite the intensive development of empirical aesthetics [39], aesthetic needs, understood as the drive for aesthetic experiences, remains neglected in scientific research. This is probably due to the fact that contemporary culture, including art, has devalued beauty and rejected aesthetic needs as a path to self-actualization and self-transcendence [9,14].

### Aim of the studies and hypotheses

In creating a working definition of aesthetic needs, we have exploratively hypothesized the existence of three domains of aesthetic needs. Our working definition: Aesthetic needs create drives to experience beauty and other aesthetic qualities/stimuli. Aesthetic needs characterize people to varying degrees and can apply to many, or only selected, aspects of the world. Aesthetic needs include: 1) the need to aestheticize everyday life (aesthetic experiences of everyday objects and events unrelated to art, such as the presentation of food or the appearance of a workspace, etc.); 2) the need for contact with aesthetic creations (the arts); 3) the need to aestheticize the built and natural environments (urban spaces, architecture, parks, wild nature, etc.). The manifestation of an individual’s pursuit of fulfilling aesthetic needs involves searching for, participating in, and/or creating opportunities to experience, contemplate, and subjectively understand beauty and other aesthetic qualities.

No measure of AN exists. Therefore, the main goal of the studies reported in this paper is to develop and validate an aesthetic needs scale.

### Study 1

H1: An EFA of a proposed measure of AN will resolve into three factors which could be rationally labeled (a) the need to aestheticize everyday life, (b) the need for contact with aesthetic creations (the arts), and (c) the need to aestheticize the built and natural environments.

**Study 2** examined the following hypotheses:

H2: A CFA will confirm H1.
H3: Aesthetic needs positively and significantly correlate with the ability to integrate beauty.

In the preceding paragraphs we pointed out beauty as crucial to reflection on aesthetics, and the repercussion of experiencing beauty may be the act to integrate it. The ability to integrate beauty is an internal transformation influenced by aesthetic experiences, which can involve contact with the arts and/or entirely ordinary events and circumstances [13]. "The conscious experience of beauty may result in a change in the way of thinking about oneself, the world, and other people, teaching one how to adopt different perspectives, to understand the emotions and behaviors of oneself and others" [30, p. 1648]. A person who sees contact with beauty as a tool for their own development will likely consider contact with beauty as a significant need for themselves. Therefore, we hypothesize that the higher an individual’s ability is to integrate beauty into their being, the greater will be their aesthetic needs.

H4: Aesthetic needs positively and significantly correlate with gratitude.

When, following Dweck [1], we presented the developmental context of needs, a person’s development always takes place in interaction with the environment, with people, and one’s own self is shaped on the basis of experiences and emotions. We were interested in the aspect of gratitude. Gratitude, in a sense that goes beyond understanding it as simply receiving something from someone, is a broader tendency of a person to feel gratitude or appreciation for what is valuable or meaningful to them [40]. Although the relationships between gratitude and aesthetic experiences and the appreciation of beauty are not well-explored areas, there is empirical evidence suggesting that grateful people are more likely to appreciate beauty [27]. For example, evaluating photographs of the beauty of the natural environment generated subsequent gratitude [41]. Additionally, gratitude and appreciation of beauty are both self-transcendent emotions/traits, and thus are theoretically related [27]. Therefore, we hypothesize that dispositional gratitude will predict levels of aesthetic needs.

H5: Aesthetic needs are positively and significantly correlated with curiosity and interest in the elements of nature.

One type of aesthetic need we have identified is the need for contact with an aesthetic environment (wider living space), including nature. It is well established that connectedness to nature is highly correlated with appreciation of nature’s beauty [42]. Likewise, curiosity and appreciation of beauty are tightly connected, especially through the personality trait of openness [43], and both load strongly on the super-virtue of inquisitiveness [44]. It is known that aesthetic quality predicts aesthetic emotions through aesthetic evaluation [45]. The level of visual pleasure regarding nature has been the focus of researchers, for example, in the context of urban forests [46]. Landscapes and also all natural phenomena, especially those that evoke a sense of awe and respect in humans, can elicit admiration, appreciation, and be perceived aesthetically. Observing the formation of a Cumulonimbus capillatus cloud (known as the blacksmith’s anvil) or perhaps any cloud formation can be an aesthetic feast for many people, and not only for "storm chasers." We hypothesize that the more that people are interested in, and curious about, natural phenomena the greater will their intensity of aesthetic needs concerning contact with nature.

H6: Aesthetic needs positively and significantly correlate with the quantity and quality of aesthetic experiences a person has with the visual arts.

Experiencing the visual arts includes emotional, perceptual, cognitive, and cultural aspects, as well as being conducive to circumstances related to, and the potential occurrence of, a state of flow [47]. We assume that individuals who exhibit a high intensity, complexity, and range of experiences, when in contact with the visual arts, will have higher aesthetic needs, especially in the dimension of needs related to contact with artistic works and events. Experiencing aesthetic dissatisfaction, through unfulfilled aesthetic needs, may foster focused and profound appreciation of art. Conversely, someone who deeply experiences contact with art and perceives it as a pleasant and enriching event will likely seek to multiply such situations. Furthermore, as the proficiency in art appreciation grows, the expectations of the recipient may become more refined, and hypothetically, the aesthetic perception could be extended to non-artistic domains of life. In such a case, the demand for aesthetic experiences might become even greater.

**Study 3** examined the following hypotheses:

H7: Aesthetic needs positively and significantly correlate with engagement with beauty.

It is difficult to discuss aesthetic needs without considering the trait and state of engagement with beauty [27]. We believe that aesthetic needs are a consequence of the universal and inherent human ability to perceive, engage with, and reflect on various forms of beauty. As there is no published empirical research linking both variables, our hypothesis is exploratory.

H8: Aesthetic needs positively and significantly predict sensitivity to disgust.

Thinking about the fact that some people may have a high need for contact with what is beautiful and/or aesthetically pleasing, we began to wonder whether such people may also experience ugliness more acutely. What is ugly can be disgusting. Disgust is a basic emotion that evolved to serve a protective function. Sensitivity to disgust indicates a predisposition to react with disgust to various, potentially dangerous, stimuli [48]. An experiment in which participants evaluated photos of various frog species revealed that people with high levels of disgust sensitivity rated frogs as less beautiful and more disgusting [49]. Another study showed that individuals more sensitive to disgust were more likely to dislike untidy rooms and asymmetric geometric patterns [50]. Therefore, we hypothesize that the higher a person’s level of aesthetic needs, the higher will be their sensitivity to stimuli potentially evoking disgust.

H9: Aesthetic needs negatively and significantly correlate with external stimulation as a dimension of boredom susceptibility.

Savoring aesthetics is associated with contemplation, deepening one’s inner life. We hypothesize that individuals seeking external stimulation through intense experiences may have lower aesthetic needs is intriguing, but direct evidence for this relationship from the research literature is lacking. However, we do know that aesthetic sensitivity, a deep engagement when experiencing art, is considered characteristic of individuals with sensory processing sensitivity and a highly sensitive personality [51,52]. The Polish version of the shortened scale for assessing highly sensitive personality also includes an item related to experiencing art or music [53]. If highly sensitive individuals (who are easily overstimulated) are more attuned to the aesthetic qualities of art or their surroundings (which would foster higher aesthetic needs), then, by analogy, people who quickly get bored and require strong external stimulation may have a lower demand for aesthetics (as they may not pay attention to subtleties, and engagement in aesthetic experiences may not be sufficiently stimulating for them).

H10: Aesthetic needs positively and significantly correlate with the need for internal stimulation as a dimension of boredom susceptibility.

Westgate and Wilson write that: “The experience of boredom motivates people to take steps toward restoring successful engagement in a meaningful activity” (p.2) [54]. In the context of Polish cultural conditions, the measure of susceptibility to boredom reveals two dimensions [55]. One of these dimensions is the level of internal stimulation, which assesses whether a person can sufficiently engage their mental resources. The perception of aesthetic stimuli, and contemplation of them, are part of an individual’s internal, psychological life. It is known that a propensity for boredom is a positive predictor of curiosity [56], and curiosity and exploration are correlates of aesthetic experiences [47]. Since it can be assumed that aesthetic needs motivate people to seek aesthetic experiences, we hypothesize that individuals with a high need for internal stimulation may also have greater aesthetic needs.

H11: Aesthetic needs are positively and significantly correlated with aesthetic response competence in art.

One of the potential components of aesthetic needs that we have identified is the need for contact with art. Although this does not necessarily mean understanding art or having extensive experience in assessing art in general, we hypothesized a relationship between the level of AN and the sophistication of the recipient of art. Dan et al. [31] point to aesthetic response competencies in various art domains as a manifestation of an Aesthetic Quotient (aesthetic intelligence). It is known that artists (individuals with high aesthetic experience, likely possessing high artistic competencies) have higher levels of engagement with artistic and natural beauty compared to non-artists [32]. If there are significant differences in the scope of engagement and experiencing beauty between artists and non-artists, then it is reasonable to hypothesize that people with high aesthetic competencies will also have greater aesthetic needs (and vice-versa; the lower the aesthetic competencies, the lower their AN).

## Study 1

Study 1 consists of an EFA, an exploratory factor analysis, of 18 potential items for the proposed Aesthetic Needs Scale (ANS).

### Method

Registered hypotheses and data from this project, for all three studies, may found at https://osf.io/96f4r/?view_only=7e296b422c664437ac5a19b018902dea

The participants in all three studies lived in Poland, and all measures were in Polish.

### Participants

Our convenience sample consisted of 184 people (69.0% women, 31% men) who were recruited via the Internet, especially such social networking websites as Facebook, LinkedIn, and Twitter. The age of the participants ranged from 18 to 83 years (*M*  =  33.97; *SD*  =  16.84). The largest number of people had completed higher education (46.2%), followed by higher education in progress (30.4%), primary education (9.8%), general secondary (8.2%), industry secondary (4.9%), and basic vocational (0.5%). In terms of place of residence, the group was diverse, but all within the nation of Poland. More participants (25.5%) gained artistic education in an informal way, learning art or music by themselves, and only 10.9% people obtained formal artistic education (e.g.: art high school or music school). As for the frequency of participation in artistic events, 53.3% participated several times a year, 13.6%–several times a month, 12.5%–once a month, 9.2%–less often or not at all, 6.5%–once a year, 4.9%–once a week or more. Among their most favorite types of artistic events, the respondents mentioned: music concerts (47.8%), theater performances (17.9%), painting and/or sculpture exhibitions (11.4%), musicals (6.5%), photo exhibitions (6.5%), opera and/or operetta (3.8%), ballet performances (2.2%), literary meetings and/or poetry evenings (1.1%). Only 2.7% of the participants marked none of the abovementioned events. All participants included in our data analysis provided written informed consent to take part in the study. The study took place in May and June 2023.

### Measure

The initial stage of developing the Aesthetic Needs Scale (ANS) involved conducting interviews with several individuals to create a working definition of aesthetic needs. Among these individuals were both art experts (e.g., formally educated in music) and laypersons (people with education and work unrelated to any artistic field), as well as a person with expertise in philosophy (aesthetics being a philosophical domain). The information obtained, firstly, allowed for the identification of three categories of aesthetic needs. Secondly, the statements made during the interviews served as the basis for formulating the scale items.

The next step in developing the scale was creating a pool of statements. The first author was responsible for crafting the statements, while the others participated in their initial evaluation. This process resulted in 28 statements, which were then sent for assessment to a psychologist, a layperson (without expertise in psychology, art, or philology), and the third had strong linguistic competence. Their task was to evaluate the validity of the generated statements, their comprehensibility, and language correctness. Based on the feedback received, 10 items were removed from the original pool because they were redundant (paraphrasing other items) or deviated too much from the essence of the construct. This left 18 statements, which were included in the pilot version of the measurement.

### Procedure and data analysis

In the first step, the relationship between manifest variables and hypothetical constructs (factors) of the Aesthetic Needs Scale (ANS) was explored through Exploratory Factor Analysis (EFA). All the ANS items were scanned for skewness and kurtosis to assess how closely their scores approached a normal distribution. We considered values less than ±2 for skewness [57] and less than ±7 for kurtosis [58] as indicators of a relatively normal distribution. The appropriate sample size was determined based on a subject-to-item ratio of 10:1, which is commonly advocated [59].

To extract factors, the maximum likelihood (ML) extraction method was used with oblique rotation (promax) as we assumed that the data would be normally distributed [60] and the three factors of the ANS would be correlated. With respect to factor retention, several criteria were followed based on the recommended best practices for EFA [61]: 1) factors with eigenvalues greater than one (Kaiser criterion); 2) a scree plot; and 3) a parallel analysis of Monte Carlo simulations relying on random number generations. The project was conducted in accordance with the Declaration of Helsinki. All statistics were analyzed using IBM SPSS statistics package version 20 and IBM SPSS AMOS 21. Moreover, parallel analysis was measured using a web utility at https://analytics.gonzaga.edu/parallelengine/ [62].

### Results

A review of the descriptive statistics illustrated satisfactory values for skewness (less than ±2) and for kurtosis (less than ±7) thus confirming near-normal distribution of all items included in the original item pool. Besides skewness and kurtosis values, means, standard deviations, minimum, maximum, and reliability coefficients for the scale, if an item was deleted, are displayed in Table 1.

**Table 1:** Study 1: *Descriptive statistics*, *Skewness*, *Kurtosis*, *and reliability coefficients if item was deleted (N = 184)*.

Items	Mean	SD	Min	Max	Skewness	Kurtosis	α if item deleted
ANS1	5.19	1.38	1	7	-0.62	0.13	0.88
ANS2	5.30	1.33	1	7	-.079	0.20	0.88
ANS3	5.39	1.39	1	7	-0.88	0.27	0.88
ANS4	5.34	1.39	1	7	-0.73	-0.01	0.88
ANS5	4.87	1.61	1	7	-0.41	-0.85	0.88
ANS6	4.88	1.50	1	7	-0.43	-0.49	0.88
ANS7	5.40	1.52	1	7	-1.05	0.62	0.88
ANS8	5.77	1.36	1	7	-1.27	1.37	0.88
ANS9	5.47	1.50	1	7	-0.85	-0.04	0.89
ANS10	5.57	1.48	1	7	-0.78	-0.36	0.88
ANS11	5.20	1.69	1	7	-0.70	-0.45	0.89
ANS12	5.24	1.56	1	7	-1.04	0.70	0.89
ANS13	5.81	1.16	1	7	-1.10	1.19	0.89
ANS14	6.04	1.27	1	7	-1.46	1.72	0.89
ANS15	5.82	1.28	1	7	-1.19	1.14	0.89
ANS16	5.68	1.30	1	7	-0.92	0.41	0.89
ANS17	5.85	1.33	1	7	-1.41	2.01	0.89
ANS18	5.93	1.13	1	7	-1.23	1.77	0.88

*Note*. ANS–Aesthetic Needs Scale.

Two tests to assess sampling adequacy, the Kaiser-Meyer-Olkin statistic (0.864) and a significant probability level smaller than *p* < 0.001 for the Bartlett’s Test of Sphericity for all eighteen items, indicated that the sample met the criteria for the application of factor analysis (χ^2^ = 1952.872, *df* = 153).

**Fig 1:**
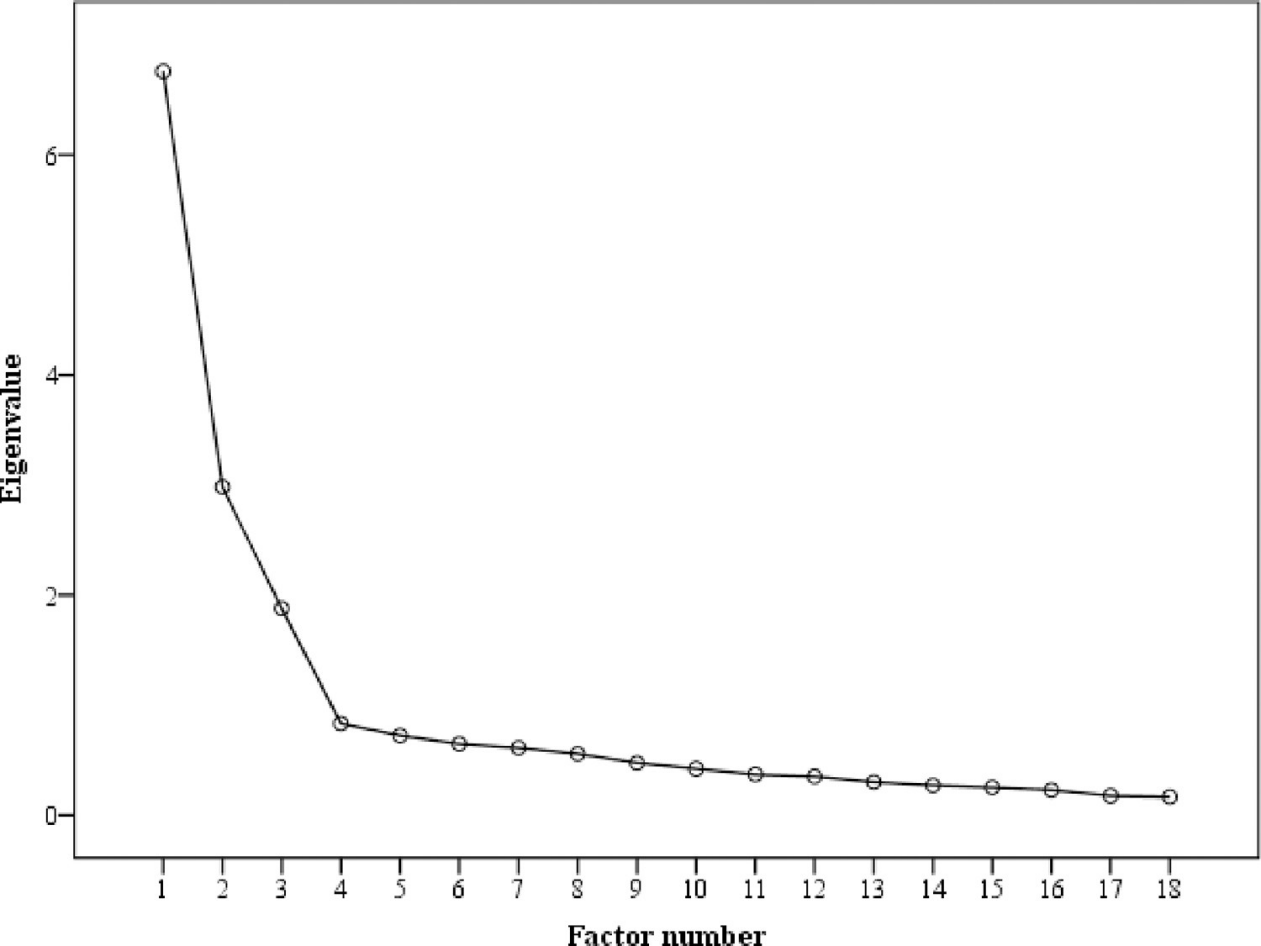
Study 1: Scree plot of original 18 items.

The pool of 18 items was subjected to EFA with maximum likelihood (ML) estimation (promax rotation). A visual scree plot, based on eigenvalues greater than 1.0, indicated a three-factor solution (Fig 1) which accounted for 57.665% of the total variance. Factor 1 accounted for 35.012%, factor 2–14.204%, and factor 3–8.448%. The outcomes of the Monte Carlo simulation with 18 items, 184 subjects, and 1,000 replications on randomly generated data, also showed a three-factor solution. In fact, from the comparison of the eigenvalues of our data set, against the eigenvalues of the random data set, the total variance explained for the three factors corresponding to actual eigenvalues, was greater than the parallel average random eigenvalues. These various techniques all suggested that three factors should be extracted.

When it came to selecting items, we followed the cut-off of 0.55 [63] to assure a more robust scale, a procedure employed within an exploratory setting, particularly when brief scales are developed [64]. Since our goal was to create a concise three-factor scale with three or four items for each factor, items ANS3, ANS4, ANS5, and ANS6 were selected for the first factor (contact with art); items ANS10, ANS11, ANS12, and ANS13 –for the second factor (aesthetics); items ANS14, ANS15, ANS16, and ANS17 –for the third factor (nature) (Table 2). In the case of the second and third factors, the choice of items is clear due to the removal of those with values lower than 0.55; with the first factor we chose the four items with the highest values, so that all factors would be represented by 4 items each.

**Table 2:** Study 1: Promax rotation (N = 184).

Items	1	Factor 2	3
ANS1	0.73		
ANS2	0.76		
ANS3	**0.85**		
ANS4	**0.79**		
ANS5	**0.80**		
ANS6	**0.89**		
ANS7	0.72		
ANS8		0.53	
ANS9		0.44	
ANS10		**0.78**	
ANS11		**0.73**	
ANS12		**0.77**	
ANS13		**0.82**	
ANS14			**0.75**
ANS15			**0.72**
ANS16			**0.58**
ANS17			**0.95**
ANS18			0.41

*Note*. ANS–Aesthetic Needs Scale.

### Discussion

H1, which stated that the initial pool of items for the ANS would resolve into three factors, was supported. A pilot version of the ANS was created containing 18 items. Results of the exploratory factor analysis showed satisfactory indicators of KMO, Bartlett’s Test, as well as Monte Carlo simulation. Based on the obtained results, the theoretically assumed 3-factor structure of the tool was affirmed. As a result, a 12-item version of the scale was created, thus allowing a CFA to be performed in Study 2, as well examining the criterion-related validity of the ANS.

## Study 2

Study 2 was focused on a confirmatory factor analysis of the 12-item ANS and addresses hypotheses H2 –H6.

### Method

#### Participants

The sample consisted of 211 people (78% women, 22% men) who, similar to Study 1, were recruited via the Internet, following the same procedure. The age of the participants was between 18 and 72 years (*M*  =  31.87; *SD*  =  15.49). Educational levels were similar to participants in Study 1. Formal artistic education was completed by 10.9% of participants and informal artistic education by 28.0%. With regard to participation in artistic events, 49.3% were involved several times a year, 13.7%–several times a month, 10.9%–once a month, 10.9%–once a year, 10%–less often or not at all, and 5.2%–once a week or more. Among the most preferred artistic events, the participants declared: music concerts (47.9%), theater performances (25.1%), painting and/or sculpture exhibitions (6.2%), musicals (4.3%), photo exhibitions (4.3%), opera and/or operetta (4.3%), literary meetings and/or poetry evenings (3.3%), and ballet performances (2.4%). “None of the abovementioned events” were marked by 2.4% of the participants in the study. All participants granted written informed consent to volunteer for this research study. The study took place in May and June 2023.

#### Measures

In the current study, a 12-item ANS was used, in line with the results obtained in Study 1. It has three subscales, each of which consists of 4 statements. The first subscale queries about contact with art (example item: “I look for information about the art that interests me.”), the second subscale relates to aesthetics needs of everyday life (example item: “The appearance and presentation of food are important to me.”), and the third addresses aesthetic needs related to outdoor spaces regarding nature and the built environment (example item: “I often look for beauty in nature.”). Since the response scale for each statement is a 7-point Likert scale (1 = "*I strongly disagree*" and 7 = "*I strongly agree*"), the respondent in a single sub-scale can receive from 4 to 28 points. The total score, as the sum of points from sub-scales, can range from 12 to 84 points. The higher the score, the greater the need for aesthetics. In Study 1, the subscale for the need of contact with art was α = 0.89; for the need of aestheticization of everyday life, α = 0.83; for the built and natural environment, α = 0.82; and total score was α = 0.83.

#### Procedure and data analysis

The main goal of the Study 2 was to verify the factor structure of the ANS that emerged from the Exploratory Factor Analysis conducted in the Study 1. This process was done through five steps: model specification, identification, parameter estimation, model evaluation, and model modification/re-estimation [65,66].

Since we assumed the presence of intercorrelations between the factors of the ANS, the variance inflation factor (VIF) and a tolerance value were computed to estimate the degree of a potential linear relationship. A cut-off of 5.0 for the VIF and a tolerance value to 0.1 were used as measures of an implicit issue of collinearity [67]. The Mahalanobis distance (χ^2^ test with respective degrees of freedom and *p* < 0.001) and Cook’s distance (close to 1) were checked to discover the existence of possible multivariate outliers.

**Fig 2:**
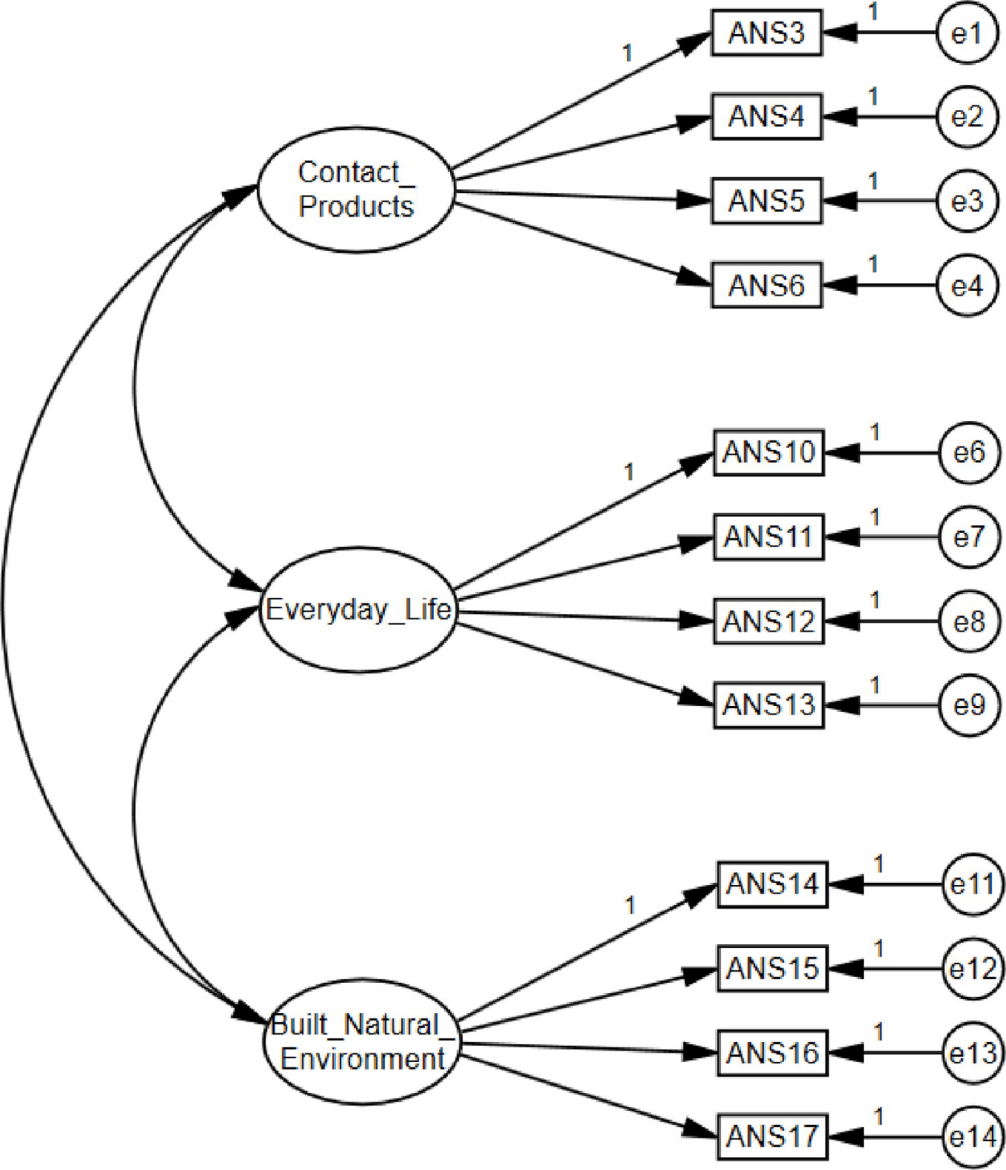
Study 1: AMOS. Hypothesized model of the ANS.

With respect to model specification, the starting point for conceptual/empirical justification of the ANS was the assumption that aesthetic needs refer to different areas of human life. Since model specification is grounded primarily in theory [68], we tried to base it on some substantive knowledge [66], recognizing that this process had a strongly exploratory character. Based on Maslow’s [2] conviction that people have aesthetic needs, we attempted to test the model of aesthetic needs characterized by three sub-constructs: 1) the need for contact with works of art (art); 2) the need to aestheticize everyday life (aesthetic experience of everyday objects and events not related to art, for example the way food is served or the appearance of the workplace); 3) the need to aestheticize the built and natural environments (nature as a source of aesthetic sensations, aesthetic aspect of urban space development, architecture). Empirical justification for the complexity of the aesthetic needs were outcomes of an exploratory analysis conducted on twelve items selected from the original 18 items of the EFA (Fig 2).

When it comes to model identification, in the first phase, the units of measure for observable and latent variables were defined. In the case of indicators (manifest variables), these were points on a Likert-type scale. In the case of latent variables, we fixed the variance of each of them to be 1.0 and set a directional path from the latent variables to indicators (Fig 2). In the second phase, we ensured that the model had nonzero degrees of freedom [69]. In fact, in this study, a model was identified based on the number of known input data being greater than the number of estimated parameters (78 > 30; *df* = 48).

In step 3, the model parameter estimates were reviewed, considering three criteria: (1) the feasibility of the parameter estimates manifested in the correct sign; (2) the appropriateness of the standard errors (small values indicating more accurate estimation); and (3) the statistical significance of the parameter estimates with critical ration (C.R.) higher than ± 1.96 [70] (Byrne, 2016). In our sample, all regression weights between observed and latent variables were significant having C.R. between 9.923 and 17.168. Hence, all manifest variables in our model were statistically significantly associated with their corresponding three key constructs (the need for contact with works of art; the need to aestheticize everyday life; the need to aestheticize the built and natural environment).

In step 4, before drawing final conclusions as to the correctness and adequacy of the model, we examined to what extent the hypothetical model of aesthetic needs with three factors, fits the empirical data. In the estimation, several of the most common goodness- and badness-of-fit indices were used: chi-square (χ^2^) with *p* insignificant value; chi-square–degrees of freedom ratio (χ^2^/*df*) ≤ 3; the Goodness-of-Fit Index (GFI), Comparative Fit Index (CFI), and Tucker-Lewis Index (TLI) ≥ 0.9; the Root Mean Square Error of Approximation (RMSEA) ≥ 0.8, its 90% Confidence Interval (HI ≤ 0.08; LO ≤ 0.05), and the Root Mean Square Residual (RMSR) ≤ 0.05 [70].

In step 5, the possibility of a potential modification/re-estimation of the model would be considered, but only in case there were unfavorable indicators of fit (although this practice is one of the most contentious aspects of Structural Equation Modeling) and having theory-based justification [69].

The bootstrapped correlation procedure was performed for 1,000 randomly formed samples for each correlation coefficient with bias-corrected accelerated limits (BCA) and 95% confidence intervals.

### Measures

In this study we used questionnaires to measure: integration of beauty (integrating experiences of beauty into the mind of the observer), dispositional gratitude, elements of nature curiosity, and aesthetic experience.

*Ability to Integrate Beauty Scale* [30] is a single-factor scale measuring Ferrucci’s [13] third pillar of aesthetic intelligence. It examines the ability to change thoughts and feelings under the influence of beauty. The tool consists of seven statements to which the respondents refer on a 7-point Likert scale (1 = *strongly disagree*, 7 = *strongly agree*); scores can range from 7 to 49. Items include “Contact with beauty prompts me to reflect,” and “Experiencing beauty teaches me a new way of looking at the world.” A high score indicates a greater ability to integrate beauty. In the present study, the Cronbach’s α was 0.93.

*Gratitude Questionnaire* [71,72] is a short, 6-item measure of gratitude as a disposition. The Polish version is congruent with the original version, psychometrically confirming the single-factor structure. Respondents assess the statements on a 7-point Likert scale (1 = *strongly disagree*, 7 = *strongly agree*); scores can range from 7 to 42. The results are summed, after reverse-scoring the two negatively formulated items. The higher score, the stronger the internal disposition to feel gratitude. In the current study, the Cronbach’s α was 0.83.

*Elements of Nature Curiosity Scale* [73] (ENCS) is an original Polish one-factor scale of 10 items that measures interest in natural phenomena. Examples of statements are: “I like watching the stormy sea” or “I like watching lightning discharges”. Items are rated on a scale from 1 –*definitely untrue* to 4 = *definitely true*; score can range from 10 to 40. The higher the score, the greater the interest, fascination, and curiosity with natural phenomena (storms, hurricanes, floods, volcanoes). In this study, Cronbach’s α was 0.83.

*Aesthetic Experience Questionnaire* [47] (AEQ) is a questionnaire that measures the dimensions of aesthetic experiences in relation to the visual arts. It consists of 22 statements that make up six factors: four artistically-related components (perceptual, emotional, cultural, and understanding) and two flow dimensions (proximal conditions and experience). Respondents assess each sentence on a 7-point Likert scale, where 1 = I strongly disagree and 7 = I strongly agree. Total score can range from 22 to 154. Example items: “I see the work of art as an extension of its time period," and “I lose track of time when I view the work of art." The Polish validation of the tool was used in this study [74]. Reliability indicators in the present study were as follow: perceptual–α = 0.84, emotional–α = 0.86, cultural–α = 0.83, understanding–α = 0.80, proximal conditions for flow–α = 0.79, flow experience–α = 0.87, and total score–α = 0.94.

### Results

Table 3 shows the descriptive statistics of all items of the ANS, factors of the ANS, AIBS, GQ-6, ENCS, and AEQ, supporting that the data are close to a normal distribution.

**Table 3:** Study 2: Descriptive statistics, Skewness and Kurtosis (N = 211).

Items	Mean	SD	Min	Max	Skewness	Kurtosis
ANS3(1)	5.24	1.56	1	7	-0.78	0.07
ANS4(2)	4.98	1.68	1	7	-.071	-0.33
ANS5(3)	4.91	1.61	1	7	-0.67	-0.23
ANS6(4)	4.74	1.63	1	7	-0.45	-0.38
ANS10(5)	5.56	1.49	1	7	-1.17	1.03
ANS11(6)	5.44	1.61	1	7	-1.12	0.51
ANS12(7)	5.45	1.50	1	7	-1.22	1.22
ANS13(8)	5.72	1.38	1	7	-1.39	2.07
ANS14(9)	6.00	1.42	1	7	-1.80	3.09
ANS15(10)	5.81	1.38	1	7	-1.47	2.09
ANS16(11)	5.61	1.46	1	7	-1.19	1.12
ANS17(12)	5.82	1.52	1	7	-1.56	2.13
ANS Contact Products	5.04	1.41	4	28	-0.75	0.28
ANS Everyday Life	5.54	1.26	4	28	-1.28	2.05
ANS Build and Natural Environment	5.80	1.25	4	28	-1.61	2.92
ANS Total	5.46	1.04	12	84	-1.54	3.99
AIBS–Beauty Integration	5.57	1.30	7	49	-1.31	1.98
GQ–Gratitude Questionnaire	5.47	1.15	6	42	-0.78	0.30
EN–Elements of Nature	2.48	0.64	10	40	-0.12	-0.40
AEQ Emotional	5.05	1.24	4	28	-0.91	0.91
AEQ Cultural	4.47	1.39	4	28	-0.54	-0.14
AEQ Perceptive	5.11	1.35	3	21	-1.06	0.96
AEQ Understanding	4.85	1.21	4	28	-0.94	1.16
AEQ Proximal Flow	4.16	1.25	3	21	-0.57	0.21
AEQ Flow experience	4.45	1.41	4	28	-0.33	-0.30
AEQ Total	4.68	1.03	22	154	-0.95	1.57

*Note*. ANS—Aesthetic Needs Scale; AIBS—Ability to Integrate Beauty Scale; GQ—Gratitude Questionnaire; EN—Elements of Nature Curiosity Scale; AEQ—Aesthetic Experience Questionnaire.

All VIF values were less than the threshold value of 5.0 (range: 1.083–2.003) and the tolerance values were between 0.499 and 0.923, indicating that multicollinearity was not a major concern in sample 2. An analysis of the Mahalanobis distance method displayed two multivariate outliers in the dataset (*p* = 0.00039 and *p* = 0.00046) both with a significance level below *p* < 0.001. Nevertheless, given that recalculation of the statistics with and without the detected anomalous cases produced very comparable outcomes, the observations with large residuals were not excluded from the analysis. The Cook’s distance values ranged between 0.000 and 0.108 further supporting that outliers were not problematic in the data.

**Fig 3:**
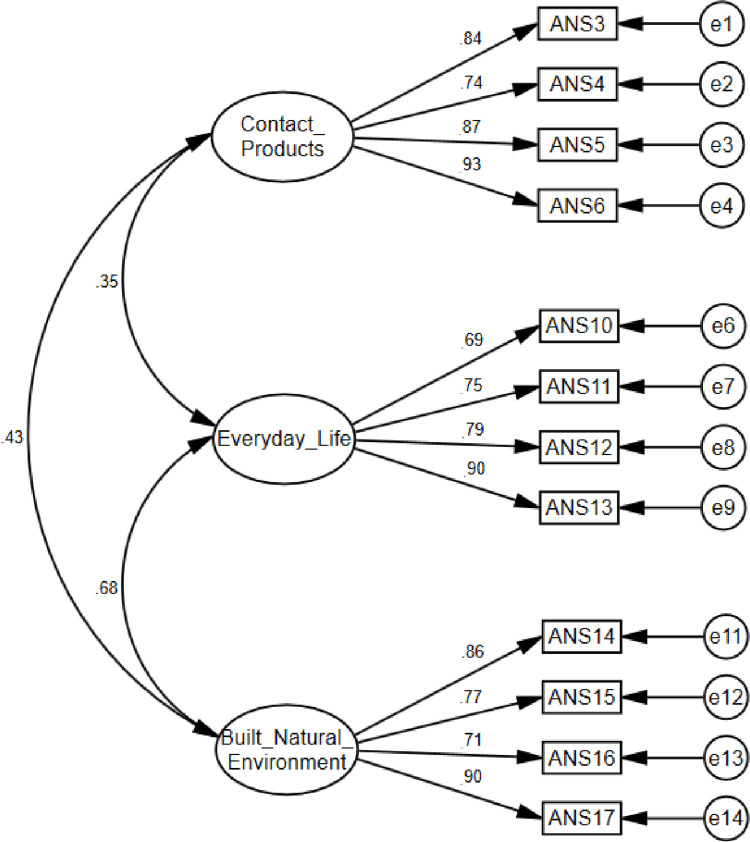
Study 2: Measurement model of final ANS.

As the EFA favored a three-factor solution, the model was specified with three latent factors: 1) the need for contact with art; 2) the need to aestheticize everyday life; 3) the need to aestheticize the built and natural environments. The factorial structure of the ANS, previously obtained in the Study 1, was confirmed through the CFA in the Study 2. The loadings were excellent (between 0.69 and 0.93) for all twelve items of the ANS (Fig 3). The goodness-of-fit showed a three-factorial solution, which well represented the entire data set: χ^2^ = 119.323, p < 0.001; χ^2^/*df* = 2.340; GFI = 0.915; CFI = 0.96; TLI = 0.95; RMSEA = 0.08, LO = 0.06, HI = 0.10; SRMS = 0.05. Although χ^2^ was significant, this often occurs with samples over N = 200, all other indices presented a very good fit. Therefore, the model is acceptable in its present form. The internal reliability of the ANS was very good: 1) the need for contact with works of art (α = 0.90); 2) the need to aestheticize everyday life (α = 0.86); 3) the need to aestheticize the built and natural environments (α = 0.88), and the total score (α = 0.89).

The pairwise correlation coefficients (Table 4) were positive and moderate between the ANS total score and its three subscales (contact with art, need to aestheticize everyday life, and need to aestheticize the built and natural environment) and the ability to integrate beauty (H3). A similar pattern of results was observed in the case of dispositional gratitude, with the exception of contact with works of art, which had only a marginally significant correlation (H4). Weaker, but still significant positive correlations were found between need to aestheticize the built and natural environments/need to aestheticize total score and with interest in the elements of nature. Insignificant correlations were between contact with works of art/need to aestheticize everyday life and interest in the elements of nature. The need to aestheticize total score, and the three subscales, were positively associated also with aesthetic experience total score and its subscales (emotional, cultural, perceptual, understanding, flow proximal conditions, and flow experience) (H6). Positive correlations were also noted between age and contact with art works (*r* = 0.20**, *p* = 0.004), need to aestheticize the built and natural environment (*r* = 0.17*, *p* = 014), and the ANS total score (*r* = 0.17*, *p* = 014).

**Table 4:** Study 2: Correlations (and CIs) between subscales and total score of ANS, AIBS, GQ, EN, AEQ (N = 211).

	ANS-C	ANS-E	ANS-BN	ANS-T	AIBS	GQ	EN	AEQ-E	AEQ-C	AEQ-P	AEQ-U	AEQ-FC	AEQ-FE	AEQ-O
ANS-C	1													
ANS-E	.376*** [.202-.532]	1												
ANS-BN	.403*** [.218-.559]	.579*** [.418-.704]	1											
ANS-T	.766*** [.680-.835]	.807*** [.734-.862]	.817*** [.731-.876]	1										
AIBS	.485*** [.347-.603]	.525*** [.391-.637]	.639*** [.513-.737]	.688*** [.592-.762]	1									
GQ-6	.115^t^ [-.030-.262]	.441*** [.319-.550]	.412*** [.297-.517]	.395*** [.274-.504]	.402*** [.291-.500]	1								
EN	.024 [-.121-.171]	.124^t^ [-.017-.267]	.253*** [.103-.391]	.162* [.033-.296]	.143* [.017-.270]	.028 [-.107-.156]	1							
AEQ-E	.529*** [.399-.652]	.464*** [.292-.602]	.510*** [.354-.634]	.631*** [.518-.723]	.597*** [.461-.710]	.410*** [.288-.516]	.127^t^ [-.023-.270]	1						
AEQ-C	.339*** [.181-.484]	.300*** [.127-.452]	.290*** [.121-.437]	.391*** [.211-.541]	.417*** [.264-.547]	.108 [-.029-.252]	.186** [.047-.313]	.344*** [.182-.498]	1					
AEQ-P	.523*** [.395-.641]	.466*** [.322-.604]	.397*** [.234-.545]	.584*** [.440-.704]	.498*** [.347-.640]	.161* [.008-.323]	.079 [-.052-.214]	.533*** [.392-.663]	.667*** [.559-.754]	1				
AEQ-U	.552*** [.421-.666]	.415*** [.242-.553]	.415*** [.235-.566]	.584*** [.455-.686]	.550*** [.402-.677]	.245*** [.104-.371]	.096 [-.038-.229]	.591*** [.475-.692]	.560*** [.435-.673]	.667*** [.536-.775]	1			
AEQ-FC	.375*** [.242-.500]	.221** [.030-.391]	.163* [-.018-.335]	.324*** [.144-.481]	.319*** [.143-.464]	.070 [-.070-.202]	.115^t^ [-.016-.252]	.390*** [.222-.525]	.478*** [.338-.597]	.536*** [.393-.648]	.582*** [.542-.689]	1		
AEQ-FE	.494*** [.365-.613]	.222*** [.116-.404]	.323*** [.159-.455]	.462*** [.318-.591]	.479*** [.342-.593]	.139* [-.010-.276]	.174* [.043-.304]	.526*** [.383-.650]	.474*** [.334-.581]	.598*** [.482-.691]	.610*** [.501-.708]	.587*** [.452-.688]	1	
AEQ-T	.593*** [.479-.699]	.450*** [.301-.577]	.442*** [.280-.580]	.628*** [.498-.734]	.604*** [.469-.710]	.236** [.089-.363]	.167* [.040-.301]	.709*** [.602-.794]	.754*** [.689-.810]	.851*** [.796-.890]	.843*** [.778-.893]	.753*** [.661-.818]	.809*** [.750-.852]	1

Note

^t^ 0.05 < p < 0.1

* p < 0.05

** p < 0.01

*** p < 0.001

ANS-C—Aesthetics Need Scale, Contact with work of art; ANS-E—Need to aestheticize everyday life; ANS-BN—Built and natural environment; ANS-T—Aesthetics Need Scale total; AIBS—Ability to Integrate Beauty Scale; GQ—Gratitude Questionnaire; EN—Elements of Nature Curiosity Scale; AEQ-E—Aesthetic Experience Questionnaire Emotional; AEQ-C—Cultural; AEQ-P—Perceptual; AEQ-U—Understanding; AEQ-FC—Flow–Proximal Conditions; AEQ-FE—Flow–Experience; AEQ-T—Total. Aesthetic Experience Questionnaire.

### Discussion

The aim of the second study was to conduct Confirmatory Factor Analysis to verify whether the three-factor structure of the questionnaire, demonstrated in Study 1 for the 12-item model, would be confirmed. The results revealed a very good fit of the model to the data, such that there was no need for its modification, and thus supporting H2. In this study, four other hypotheses (H3—H6) were also formulated and were all confirmed.

The third hypothesis (H3) concerned the positive correlation between aesthetic needs (particularly the aesthetics of art subscale) and the ability to integrate beauty [13], and the results incrementally confirmed the validity of the ANS. That individuals with a high demand for aesthetic stimuli also possess a higher ability to integrate beauty provides indirect evidence of the developmental role of contact with beauty in human life.

Hypothesis H4 was the assumption that the disposition for gratitude is positively related to aesthetic needs. Results are consistent with the theoretical reports of other researchers [40], suggesting that beauty can be a source of gratitude and that gratitude promotes the appreciation of beauty. Thus, the realization of aesthetic needs can potentially lead to increased positive emotional experiences and well-being. Gratitude is strongly linked to well-being [75] and is a self-transcendent emotion [76]. Therefore, it can be inferred that fulfilling aesthetic needs contributes to mental health.

Hypothesis H5 indicated a significant positive correlation between the ANS subscale for aesthetic needs related to nature and the built environments and an interest in, and curiosity about, natural phenomena; and this was confirmed. Although it has been shown that appreciation of nature’s beauty predicts feeling connected to nature [77], this is the first time that data have shown that interest in and curiosity about nature may fulfill aesthetic *needs*. A marginally significant correlation was also noted between an interest in natural phenomena and the aesthetics in everyday life subscale, and the aesthetics subscale related to art had a very low, non-significant correlation, thus showing discriminant validity for the ANS subscales.

Hypothesis (H6) stated that aesthetic needs (especially the subscale related to contact with art) are positively correlated with aesthetic experiences when viewing visual art. The results confirmed the assumption that individuals who more deeply and intensely experience the emotional, perceptual, cognitive, and cultural aspects of visual art, as well as experience more flow when viewing art [47], also have higher aesthetic needs across all three subscales of the ANS. The finding adds to the convergent validity of the ANS. It indicates that a high demand/need for aesthetics in various life domains may sensitize individuals to the experience of artistic works.

## Study 3

The purpose of the third study was to perform the CFA for the 12-item ANS on a new sample, independent of the two previous ones, to ensure that the model fit the data. In addition, we examined correlations of the ANS with related constructs, such as engagement with beauty and aesthetic competence, in order to further assess criterion-related validity. We also explored the relationships between the need for aesthetics and sensitivity to disgust; as well as the ANS and the need for stimulation, in the hope it would shed further illumination on the nature of aesthetic needs.

### Method

#### Participants

Participants consisted of 197 individuals (59.1% were women, 39.1% were men, and 1.5% identified as other). The participants were recruited from internet users, and the questionnaire was distributed through social media platforms such as Facebook and Twitter using links, as well as through direct contact using QR codes.

The age of the respondents ranged from 18 to 77 years (*M* = 26.26; *SD* = 11.09). The largest group of participants had completed higher education (35%), followed closely by those currently pursuing higher education, i.e., college or university students (34%); the remaining education levels of the study participants were very similar to our Studies 1 and 2.

Among the surveyed individuals, 93.9% of the participants had education and/or work unrelated to any artistic field, while only 12 individuals (6.1%) declared having formal education and/or work related to art creation, sales, or preservation. In response to the question about the frequency of attending artistic events (such as concerts, theater performances, art exhibitions, etc.), 39.1% of the respondents stated that they participate in such events several times a year. 26.9% of the participants attend artistic events less than once a year or never, 12.2% engage with artistic events once a month, 10.7% do so several times a month, 8.6% are participants at artistic events about once a year, and only 2.5% of the survey participants declared attending artistic events once a week or more frequently.

All participants included in our data analysis provided written informed consent to take part in the study. Data collection took place between 15^th^ and 30^th^ July 2023.

#### Measures

*Aesthetic Needs Scale* (ANS). See Study 2 for a description of the ANS. The reliability coefficients (Cronbach’s alpha) in Study 3 were: α = 0.87 for everyday life, α = 0.89 for the need for contact with art, α = 0.91 for aesthetics in the built and natural environments, and α = 0.92 for the entire scale.

*Engagement with Beauty Scale—Revised* [78] (EBS-R). This scale measures the level of engagement with beauty. Similar to the original version [27] (EBS), the EBS-R allows for the assessment of perceiving and appreciating beauty separately in relation to nature, art, and morality. Additionally, the revised scale includes an additional fourth dimension/subscale, which is engagement with beautiful ideas. The tool consists of 18 items, and participants rate their agreement with each statement on a 7-point Likert scale, from 1 (*very unlike me*) to 7 (*very much like me*) on questions such as “When perceiving beauty in nature I feel emotional, it ‘moves me,’ such as feeling a sense of awe or wonder or excitement or admiration or upliftment,” and “When perceiving beauty in a work of art I feel changes in my body, such as a lump in my throat, an expansion in my chest, faster heart beat, or other bodily responses.” The Engagement with Moral Beauty subscale scores range from 6 to 42, and the other three subscales range from 4 to 28; total score from 18 to 126. A higher total score indicates a greater capacity for engagement with beauty. The tool has been translated into Polish. The lead authors of this paper initially translated the English-language version to Polish; it was also submitted to independent professional translators. We then prepared a coherent Polish version by comparing our translation to the professional translators’ version.

In the current study, the alphas were: α = 0.86 for engagement with the beauty of nature, α = 0.87 for engagement with artistic beauty, α = 0.92 for engagement with moral beauty, α = 0.89 for engagement with ideational beauty, and α = 0.94 for total score.

*Questionnaire for the Assessment of Disgust Sensitivity* [34,48] (QADS). The Polish adaptation of this questionnaire was designed to measure the intensity of disgust sensitivity in three dimensions: Core Disgust, Animal-Reminder, and Contamination-Interpersonal. It consists of 37 items that are grouped into these three subscales. Participants rate their agreement with each statement using a 5-point Likert scale (how unpleasant is the given situation from 1 = "*almost unpleasant*" to 5 = "*very unpleasant*". Example statements: "You smell vomit.", "You see insect larvae on a piece of meat in a dumpster outside the house." or "You touched a dead person." The scores are summed, and a higher score indicates a greater susceptibility to experiencing disgust.

For our purposes, only the subscale for general disgust (Core Disgust) was utilized. Total score for this subscale can range from 15 to 75; the alpha in the current study was α = 0.92.

*Boredom Proneness Scale* [55,79] (BPS). This scale is used to measure the need for stimulation. In our study, the validated Polish version was utilized, which consists of 12 items [55]. Participants rate their agreement with each statement on a 7-point Likert scale from 1 = “*I strongly disagree*” to 7 = “*I strongly agree*”). Example statements: "If I’m not doing something exciting or even dangerous, I feel barely alive and numb." or "I often wake up with new ideas." The scores obtained are summed within two subscales: Internal Stimulation and External Stimulation. The total score allows for an estimation of an individual’s level of capacity to provide themselves with a satisfying level of internal and environmental stimulation, the lack of which can be perceived as a state of boredom. In each of the two subscales scores range from 6 to 42 points, and the total score can range from 12 to 84.

In the current study, the reliability coefficients (Cronbach’s alpha) were: α = 0.72 for the internal stimulation dimension, α = 0.80 for the external stimulation dimension, and α = 0.80 for total score.

*Aesthetic Competence Scale* [31] (ACS). This scale measures an Aesthetic Quotient (aesthetic aptitude or aesthetic intelligence), understood as competencies for understanding four different artistic domains: music, visual arts, literature, and film. The measurement comprises a total of 20 statements, which belong to four factors. Example statements: for the music—“I can make out one or more performing instruments when listening to music.”, for the drawing—“I can identify the style and genre of a painting.”, for literature—“When reading a literary work, I not only pay attention to the plots of the story but also to the beauty of the language.”, and for the film—“Movies can influence people’s values.” Participants respond to each statement and choose an answer on a 5-point Likert scale (where 1 = “*I strongly disagree*” and 5 = “*I strongly agree*”). Scores for each subscale and the total score are obtained by summing the points marked by the participants. Subscales’ scores can range from 5 to 25; total score, 20–100. A higher score indicates greater aesthetic response competencies in the respective artistic domains.

In our study, the validated Polish version of the scale was used (Świątek & Szcześniak, 2023b). In the current study, the alphas were: α = 0.82 for music, α = 0.86 for visual arts, α = 0.87 for literature, α = 0.90 for film, and α = 0.92 for total score.

### Results

Table 5 presents the descriptive statistics of all items of the ANS, subscales of the ANS, factors of the EBS–R, QADS Core disgust, factors and total score of the BPS, and factors and total score of the ACS confirming that the data are close to a normal distribution.

**Table 5:** Study 3: Descriptive statistics, Skewness, and Kurtosis (N = 197).

Items	Mean	SD	Min	Max	Skewness	Kurtosis
ANS3(1)	4.85	1.92	1	7	-0.59	-0.86
ANS4(2)	4.60	1.94	1	7	-0.43	-0.97
ANS5(3)	4.15	1.92	1	7	-0.14	-1.08
ANS6(4)	4.24	2.06	1	7	-0.25	-1.22
ANS10(5)	5.12	1.84	1	7	-0.87	-0.32
ANS11(6)	5.02	1.79	1	7	-0.77	-0.41
ANS12(7)	4.93	1.80	1	7	-0.64	-0.60
ANS13(8)	5.48	1.70	1	7	-1.17	0.48
ANS14(9)	5.57	1.74	1	7	-1.19	0.49
ANS15(10)	5.15	1.84	1	7	-0.85	-0.38
ANS16(11)	5.18	1.80	1	7	-0.86	-0.23
ANS17(12)	5.40	1.78	1	7	-1.12	0.27
ANS Contact Products	4.46	1.72	4	28	-0.41	-0.81
ANS Everyday Life	5.13	1.51	4	28	-0.86	0.02
ANS Built and Natural Environment	5.32	1.59	4	28	-1.09	0.37
ANS Total	4.97	1.34	12	84	-0.82	0.18
EBS–R Nature	4.67	1.62	4	28	-0.50	-0.60
EBS–R Art	4.28	1.62	4	28	-027	-0.81
EBS–R Morality	4.58	1.55	6	42	-0.43	-0.48
EBS–R Beautiful ideas	4.37	1.63	4	28	-0.27	-0.71
EBS–R Total	4.48	1.35	18	126	-0.42	-0.17
QADS Core Disgust	3.66	0.88	15	75	-0.79	0.26
BPS I	4.20	1.20	6	42	-0.28	-0.39
BPS E	3.56	1.40	6	42	0.40	-0.31
BPS Total	3.88	1.08	12	84	-0.09	0.03
ACS Music	3.31	0.96	5	25	-0.48	-0.17
ACS Visual arts	3.32	1.04	5	25	-0.46	-0.40
ACS Literature	3.86	0.98	5	25	-1.11	0.92
ACS Film	4.12	0.89	5	25	-1.80	3.59
ACS Total	3.65	0.76	20	100	-1.28	2.52

*Note*. ANS–Aesthetic Needs Scale; EBS-R–Engagement with Beauty Scale Revised; QADS–Questionnaire for Assessment of Disgust Sensitivity; BPS–Boredom Proneness Scale; ACS–Aesthetic Competence Scale.

All VIF values scored between 1.141 and 3.447 and the range of the tolerance values was between 0.323 and 0.876, showing that all explanatory variables in the multiple regression model did not present a strong degree of correlation in this third sample. The Mahalanobis distance indicated the occurrence of four outliers in the dataset (*p* = 0.00001, *p* = 0.00020, *p* = 0.00058, and *p* = 0.00069) with significant and conservative probability estimate *p*–values. Since the statistics calculated with and without outliers were almost identical, these four observations were not deleted. The Cook’s distance values ranged between 0.000 and 0.091 thus confirming that outlying observations were not influential in the data.

**Fig 4:**
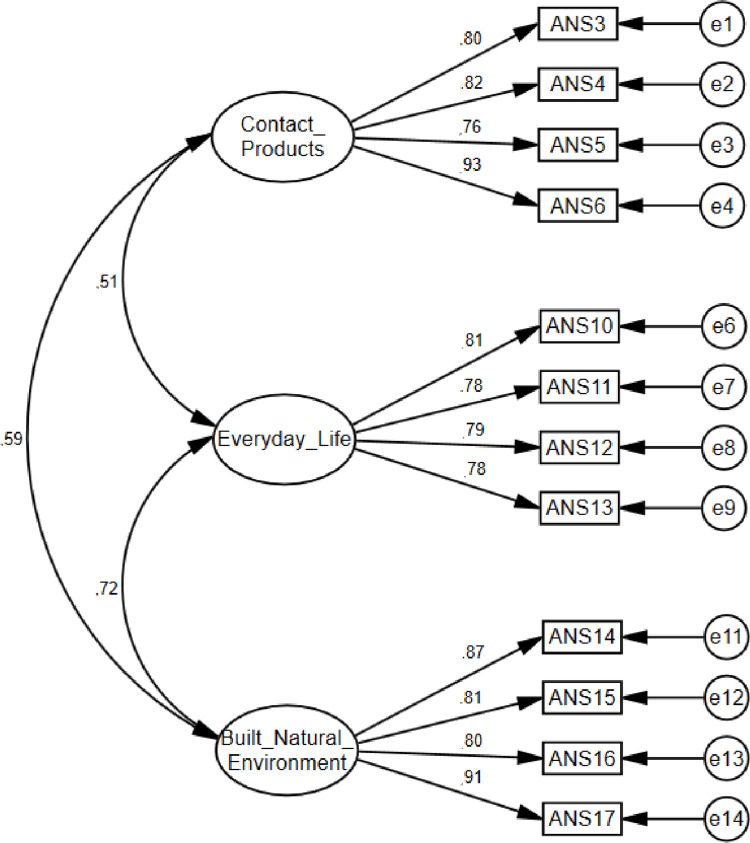
Study 3: *Measurement model of final ANS*.

The CFA analysis shows that the factor structure of the ANS was confirmed, showing results slightly better than the outcomes obtained in Study 2. The loadings were very good (between 0.76 and 0.93) for all items of the ANS (Fig 4).

The test of the goodness-of-fit provides a three-factorial solution with good fit indices: χ^2^ = 106.227, *p* < 0.001; χ^2^/*df* = 2.083; GFI = 0.92; CFI = 0.97; TLI = 0.95; RMSEA = 0.07, LO = 0.05, HI = 0.09; SRMS = 0.04. The model is satisfactory in its existent structure. The Cronbach’s alphas for the total ANS, and its three subscales, were very good: 1) the need for contact with works of art (α = 0.89); 2) the need to aestheticize everyday life (α = 0.87); 3) the need to aestheticize the built and natural environments (α = 0.91), and total score (α = 0.92).

The Pearson correlations (Table 6) showed significant positive small and moderate correlations between the total score for need to aestheticize and its subscales (contact with art, need to aestheticize everyday life, and need to aestheticize the built and natural environments) and engagement with beauty with respect to nature, art, morality, and beautiful ideas (H7). Positive and weak associations were noticed between the need to aestheticize total score/its dimensions and disgust sensitivity (H8). The need to aestheticize also correlated positively and weakly with extrinsic boredom (H9) and positively and moderately with intrinsic boredom (H10); thus the results did not confirm H9. As to H11, aesthetic needs were positively and significantly correlated with aesthetic response competence in aspects of music, visual arts, literature, film, and aesthetic response competence total score. Positive correlations were also noted between age and contact with art works (*r* = 0.19**, *p* = 0.009) and the ANS total score (*r* = 0.17*, *p* = 014).

**Table 6:** Study 3: Correlations (with confidence intervals) between dimensions/total score of ANS, EBS-R, QADS, BPS, ACS (N = 197).

	ANS-C	ANS-E	ANS-BN	ANS-O	EBS-R N	EBS-R A	EBS-R M	EBS-R I	QADS	BPS I	BPS E	BPS O	ACS M	ACS VA	ACS L	ACS F
ANS-C	1															
ANS-E	.460*** [.327-.581]	1														
ANS-BN	.531*** [.402-.646]	.642*** [.532-.736]	1													
ANS-O	.811*** [.745-.863]	.827*** [.772-.872]	.864*** [.819-.899]	1												
EBS-R N	.531*** [.417-.634]	.460*** [.328-.584]	.663*** [.569-.738]	.662*** [.574-.740]	1											
EBS-R A	.642*** [.547-.723]	.429*** [.295-.556]	.412*** [.277-.538]	.599*** [.485-.696]	.702*** [.605-.795]	1										
EBS-R M	.427*** [.277-.549]	.279*** [.123-.427]	.360*** [.216-.488]	.430*** [.265-.568]	.500*** [.355-.622]	.494*** [.362-.616]	1									
EBS-R I	.441*** [.295-.570]	.353*** [.192-.499]	.437*** [.278-.581]	.494*** [.324-.640]	.563*** [.438-.682]	.573*** [.450-.691]	.803*** [.734-.859]	1								
QADS	.251*** [.095-.396]	.278*** [.147-.407]	.278*** [.135-.402]	.322*** [.178-.453]	.266*** [.124-.393]	.251*** [.106-.395]	.241** [.098-.358]	.203** [.052-.337]	1							
BPS I	.503*** [.391-.608]	.348*** [.209-.473]	.406*** [.260-.537]	.506*** [.379-.621]	.510*** [.402-.614]	.491*** [.364-.603]	.325*** [.147-.489]	.399*** [.252-.537]	.216** [.052-.391]	1						
BPS E	.167* [.017-.322]	.129^t^ [-.033-.277]	.167* [.015-.311]	.186** [.029-.338]	.206** [.041-.372]	.235** [.070-.384]	.139^t^ [-.015-.300]	.230** [.072-.373]	.137^t^ [-.009-.272]	.380*** [.229-.523]	1					
BPS O	.387*** [.256-.513]	.277*** [.122-.415]	.333*** [.175-.484]	.401*** [.245-.539]	.417*** [.283-.544]	.425*** [.293-.543]	.270*** [.121-.437]	,371*** [.218-.517]	.208** [.044-.368]	.801*** [.729-.853]	.858*** [.819-.893]	1				
ACS M	.451*** [.315-.576]	.257*** [.094-.421]	.229*** [.081-.382]	.380*** [.228-.527]	.309*** [.166-.442]	.420*** [.290-.539]	.195** [.042-.346]	.278*** [.129-.417]	.071 [-.104-.242]	.352*** [.204-.486]	.211** [.062-.358]	.332*** [.176-.478]	1			
ACS VA	.561*** [.437-.655]	.474*** [.351-.590]	.496*** [.354-.620]	.615*** [.509-.708]	.555*** [.442-.656]	.635*** [.546-.716]	.346*** [.204-.473]	.420*** [.262-.553]	.230** [.070-.396]	.450*** [.299-.571]	.192** [.033-.342]	.374*** [.225-.500]	.418*** [.279-.553]	1		
ACS L	.548*** [.422-.647]	.423*** [.269-.574]	.517*** [.376-.628]	.598*** [.471-.701]	.527*** [.405-.638]	.497*** [.367-.608]	.480*** [.343-.608]	.458*** [.319-.573]	.274*** [.094-.440]	.517*** [.403-.620]	.083 [-.087-.260]	.341*** [.194-.486]	.361*** [.197-.513]	.580*** [.448-.694]	1	
ACS F	.495*** [.374-.592]	.434*** [.268-.572]	.541*** [.398-.650]	.589*** [.457-.688]	.456*** [.328-.571]	.451*** [.327-.573]	.371*** [.203-.512]	.379*** [.221-.510]	.262*** [.045-.442]	.460*** [.316-.586]	.203** [.050-.356]	.387*** [.230-.528]	.411*** [.210-.570]	.499*** [.338-.624]	.643*** [.502-.756]	1
ACS O	.658*** [.559-.730]	.508*** [.375-.635]	.568*** [.446-.675]	.697*** [.595-.783]	.593*** [.502-.674]	.643*** [.569-.713]	.445*** [.284-.580]	.491*** [.325-.623]	.267*** [.061-.454]	.568*** [.449-.668]	.219** [.048-.379]	.457*** [.313-.586]	.696*** [.602-.774]	.808*** [.751-.858]	.826*** [.755-.876]	.803*** [.710-.870]

Note

^t^ 0.05 < p < 0.1

* p < 0.05

** p < 0.01

*** p < 0.001

ANS-C—Contact with work of art; ANS-E—Need to aestheticize everyday life; ANS-N—Built and natural environment; ANS-T—Need to aestheticize total; EBS-R N—Engagement with Beauty Scale Nature; EBS-R A—Art; EBS-R M—Morality; EBS-R I—Beautiful ideas; QADS—Disgust sensitivity; BPS I—Intrinsic boredom; BPS E—Extrinsic boredom; BPS O—Boredom total; ACS M—Aesthetic Competence Scale Music; ACS VA—Visual arts; ACS L—Literature; ACS F—Film; ACS O—Total.

### Discussion

In this study, CFA was once again conducted to examine the fit of the data to the model on a new, independent sample. All indicators showed highly satisfactory results, and based on these indicators, the Aesthetic Needs Scale (ANS) can be considered a reliable and well-structured measure of aesthetic needs. Additionally, five hypotheses, H7 –H11, were tested to assess the validity of the tool and provide insights into the relationships between aesthetic needs and other constructs and to address criterion-related validity.

Hypothesis H7 was based on the idea that those with the highest levels of trait appreciation of beauty are likely also have the highest levels of aesthetic needs [14,27]; thus we expected trait engagement with beauty to predict levels of aesthetic needs. The results confirmed this assumption, indicating significant moderate to large positive correlations between the subscales of both tools. This can be interpreted as suggesting that individuals strongly engaged in experiencing four forms of beauty, that is, natural beauty, artistic beauty, moral beauty, and appreciation of beautiful ideas [78] also possess higher aesthetic needs. This finding further demonstrates convergent validity for the ANS. If, according to Dweck [1], who we are (our personality characteristics) depends on the motivation to meet basic and emergent needs, then aesthetic needs can shape the personality trait of engagement with beauty.

Hypothesis H8 concerned the relationship between aesthetic needs and sensitivity to disgust. Although Rabb et al. [80] found that inducing a *state* of disgust did not influence aesthetic judgement (but did influence moral judgment), no research publication has reported on the relationship between *trait* disgust and aesthetic needs. We theorized that individuals with high aesthetic needs are also more sensitive to potentially ugly and disgusting experiences [34,48]. Indeed, our data show this is the case, thus also showing more criterion-related convergent validity for the ANS.

Two hypotheses related to boredom were also formulated (H9 and H10). Theoretically, and empirically, aesthetic sensitivity, a deep engagement when experiencing art, is considered characteristic of individuals with sensory processing sensitivity and a highly sensitive personality [51,52]. Therefore, we expected aesthetic needs to be positively correlated with the susceptibility to boredom. In particular, we hypothesized that those with a high demand for internal stimulation, would have higher levels of aesthetic needs (H10), as fulfilling aesthetic needs can provide material for pleasant, reflective contemplation. And, indeed, this is what we found, thus demonstrating further criterion-related convergent validity for the ANS. However, hypothesis H9 predicted a negative correlation between aesthetic needs and external stimulation because we theorized that people who require strong external stimulation may have a lower demand for aesthetics (as engagement in many aesthetic experiences may be too subtle and mellow for them, not be sufficiently stimulating). Our data show we were wrong; although we had the trend correct. The three subscales of the ANS had *r*s of .35 to .50 with boredom/internal stimulation; and they had *r*s of only 13 to .17 with boredom/external stimulation. In retrospect, although many (most?) aesthetic experiences are primarily considered as internal stimulation (e.g., individual contemplation of nature or quietly observing an artwork in a museum or listening to a largo), some ways of fulfilling aesthetic needs may be highly stimulating. Participating in a heavy metal concert with a large audience or some forms of street performances, for example, are potentially highly stimulating for both performers and the audience.

Hypothesis H11 focused on examining the relationship between levels of aesthetic needs and levels of competence in appreciating and understanding artworks in the domains of music, visual arts, literature, and film [31]. The results support the hypothesis, showing that individuals with higher level skills in the appreciation of a wide range of artworks, generally have a higher need for aesthetic experience in various areas of their lives; in fact the correlation between the total scores on the ANS and the ACS was very large at .70. This can be explained by considering that fulfilling aesthetic needs through engagement with art (frequency of engagement, intensity of engagement, range of artworks engaged) would set the conditions to enhance *competencies* in understanding and appreciating artworks.

## General discussion

The aim of this article was to draw attention to the role and importance of aesthetic needs in human development and to present the process of developing a measurement tool for assessing individual differences in levels of aesthetic needs (the final version of the scale is in the S1 Appendix). Three separate factor analyses confirmed that aesthetic needs can be rationally and empirically framed in three dimensions: 1) the need to aestheticize everyday life (aesthetic experiences of everyday objects and events unrelated to art, such as the presentation of food or the appearance of a workspace, etc.); 2) the need for contact with aesthetic creations (the arts); 3) The need to aestheticize the built and natural environments (urban spaces, architecture, parks, wild nature, etc.). The Aesthetic Needs Scale was shown to have good internal consistency reliability. A variety of criterion-related validity studies demonstrated its convergent validity with measures of (a) integrating beauty experiences into the mind, (b) gratitude, (c) curiosity about nature, (d) emotional, perceptual, cognitive, and cultural aspects of contact with art, as well as flow, (e) engagement with and appreciation of natural beauty, artistic beauty, moral beauty, and beautiful ideas, (f) sensitivity to disgust, (g) boredom and internal stimulation, and (h) competence with interpreting and understanding works of multiple domains of art. The ANS is a first attempt to capture aesthetic needs as individual differences and it appears it is worthy of future use in other studies.

The translation from Polish to English went smoothly, with no particular difficulties involved. The concepts in the questionnaire’s items are fairly common and have direct equivalents in English. This was confirmed by both the professional translators, and the psychologists (the two lead authors) who carefully examined the translation. It is anticipated that the Polish version could relatively easily be translated into most European languages.

Maslow [2] wrote, several decades ago, that we know less about aesthetic needs than any of the other humans needs; and that has not changed. No psychologist has published a work that focused directly on aesthetic *needs*. And despite Maslow writing that the craving for beauty, and the fulfillment of aesthetic needs “is seen almost universally in healthy children.” (p. 25), aesthetic needs are not even mentioned in Dweck’s [1] Unified Theory of human development. Needs, and the fulfillment of them are the basic building blocks of development and flourishing [1]. Can we flourish without fulfilling aesthetic needs? It seems unlikely [14].

We theorized that the need for aesthetic experience is an aspect of Dweck’s [1] self-coherence needs. Fulfilling those needs creates a sense of wholeness in a person, a feeling of the personality being unified, a sense of meaning in life attained, and developmental flourishing. We believe that fulfilling aesthetic needs is essential for such wholeness and flourishing. Although we show no empirical evidence of this in our studies reported here, we consider it a very important topic for future research.

Dweck stated that for any true need “there is [a] chronic, high, and *universal value* attached to the goals that serve it” [1, p. 690; italics added]. The universal value of aesthetic needs may well be a life of contact with beauty, with integrating beauty into our being [13]; as Danto [9, pp. 14–15; italics added] argued, “Beauty is … a virtue, like truth and goodness. It is not simply among the *values* we live by, but one of the *values* that defines what a fully human life means”. Dweck also explained that achieving the goals of needs are critical “for optimal well-being in the present and optimal psychological development in the future” [1, p. 690]. Appreciation of, and engagement with, beauty leads to both individual and collective well-being [42]; and may be essential for *optimal* well-being.

The most agreed-upon definition of beauty by Western philosophers, over the last 2.5 millennia, is *unity-in-diversity*; this includes such philosophers as Plato, Augustine, Plotinus, Ficino, Hutcheson, Dewey, Santayana, Croce, Langer, and Murdoch [77]. What is *coherency*, but unity-in-diversity? The person meeting their self-coherency needs has unified the diverse elements of their personality into an integrated whole, a flourishing identity. And that is a beautiful being. Thus, we propose that fulfilling our need for contact with beauty, and human flourishing, are causally bi-directional. Flourishing will cause us to notice and engage with our aesthetic needs [14], and fulfilling our aesthetic needs makes for a self-coherent and beautiful human being. As well-being deteriorates across our planet, and our lives become more fragmented; as we descend into materialism, as we seek power over others instead of for others—might not fulfilling our need for integrating beauty into our beings be the cure?

### Limitations and future research

Clearly work on aesthetic needs is an important area for further exploration; our studies reported here are only a small beginning. Discovering their significance for psychological growth, maintaining or regaining psychological well-being through fulfilling aesthetic needs is within our future research plans.

Regarding the scale itself, we did not assess its temporal stability. As the Aesthetic Needs Scale (ANS) measures not a trait but the level of needs, which potentially can vary depending on the circumstances in which the participants find themselves, we anticipate that test-retest reliability may not be high. On the other hand, examining the extent to which the level of aesthetic needs (both overall and in specific areas) is stable versus variable over time for individuals, considering various factors (such as stress, interests, professional engagement, or amount of leisure time), is a worthwhile direction for longitudinal research. Also, we focused primarily on criterion-related convergent validity; future studies should further examine the ANS’s discriminant validity as well.

Given the three-factor structure of the scale, another potential avenue of future research would involve cluster analysis to identify profiles of individuals with specific configurations of aesthetic needs. Investigating differences among individuals belonging to distinct profiles is a way of using the Aesthetic Needs Scale to identify different sets of aesthetic goals that may be recommended to particular persons for more effectively enhancing the fulfillment of their needs, and thus their well-being and flourishing.

There are also fascinating constructs that could be justifiably included in the validation study, but due to concerns about participants’ patience and long questionnaire batteries, we had to defer them to the future or leave them for other researchers. For instance, it would be worthwhile to utilize the ANS in studies on the conditions of artistic creativity—how much the need for contact with the aesthetic motivates independent art creation or the transformation and beautification of surroundings. Another avenue of research would be to examine the extent to which daily choices and behaviors related to aesthetic valuation (e.g., art consumption) are driven by aesthetic needs versus other factors (e.g., the desire to make an impression on others). These possibilities indicate that the ANS opens the door to numerous potential research directions that can provide deeper insights into the intricate relationship between aesthetic needs and aesthetic experiences.

The Desire for Aesthetics Scale [37] (DFAS) was developed in an American cultural milieu; and the ANS in a Polish cultural milieu. Although, as we noted above, needs and desires can be very different [38], there may be some overlap between them. Despite the word *need* being only mentioned once in the scale development paper of the DFAS [37], there may be some empirical overlap between the DFAS and the ANS; translating the DFAS to Polish, and comparing it to the ANS, is warranted.

While psychological needs are often considered to be universal across humanity [1] (Dweck, 2017), it’s important to acknowledge that the ANS was developed in a specific cultural context (Central Europe) and our samples in our three studies were all of Polish nationality. At this point, it’s challenging to determine whether, and to what extent, cultural factors will impact the psychometric properties of this tool in studies involving different populations. Therefore, we encourage further testing and sharing of results to gain a better understanding of how the ANS might perform across various cultural and national contexts.

As we conclude with the limitations of this study, we would like to consider that trying to explore the human desire to surround ourselves with what is aesthetic (i.e. what is beautiful), we acknowledge the potential influence of various non-aesthetic factors. There are human motives for searching for aesthetics in various areas of life that go beyond the perceptual. Beauty can be (and is) associated with status, prestige, and well-being, as shown in advertisements for luxury goods and services. In addition, we live in a visual culture where communication through social media is increasingly based on visual content rather than verbal ("a picture is worth a thousand words"). Beauty, understood superficially, may be currently interesting to people to the extent that it can be materialized and used as a tool for individual success and profit. In our studies here, we clearly defined our view on aesthetic needs and beauty by referring to specific sources, and aiming at pure aesthetic appreciation. Other potential underlying motivations, in the search for beauty, should be the subject of further research.

### Conclusion

Our three studies present the process of constructing and validating an original scale for measuring aesthetic needs. The conceptualization of aesthetic needs and their developmental role in human life was inspired by the classic theory of needs by Maslow (1986) and the encompassing Unified Theory of Dweck [1], as well as the concept of aesthetic intelligence by Ferrucci [13]. As a result of this work, a 12-item questionnaire was developed, enabling the assessment of the intensity of aesthetic needs in three distinct aesthetic domains, as well as a total score. The results indicate that aesthetic needs are related to aesthetic experiences in art, aesthetic response competencies, engagement with four forms of beauty, the ability to integrate beauty, gratitude, interest in natural elements, sensitivity to disgust, and the demand for internal and external stimulation.

### Coda

DESIDERIO DI BELLEZZA

Asmara, 5 luglio 1955

Fervore d’idee

s’agita nel cuore

desiderio di bellezza

armonia perfezione.

Julio Savi [81, p. 5]

YEARNING FOR BEAUTY

Asmara, 5 July 1955

Fervour of ideas

is seething in the heart

yearning for beauty

harmony perfection.

Julio Savi [82, p. 5]

## Supporting information

S1 Appendix (TIF)
