# Supplementary figures and images for: The unexplored territory of aesthetic needs and the development of the Aesthetic Needs Scale
Source: PLoS One. 2024 Mar 18;19(3):e0299326. doi: 10.1371/journal.pone.0299326 (PMC10947697; doi:10.1371/journal.pone.0299326)

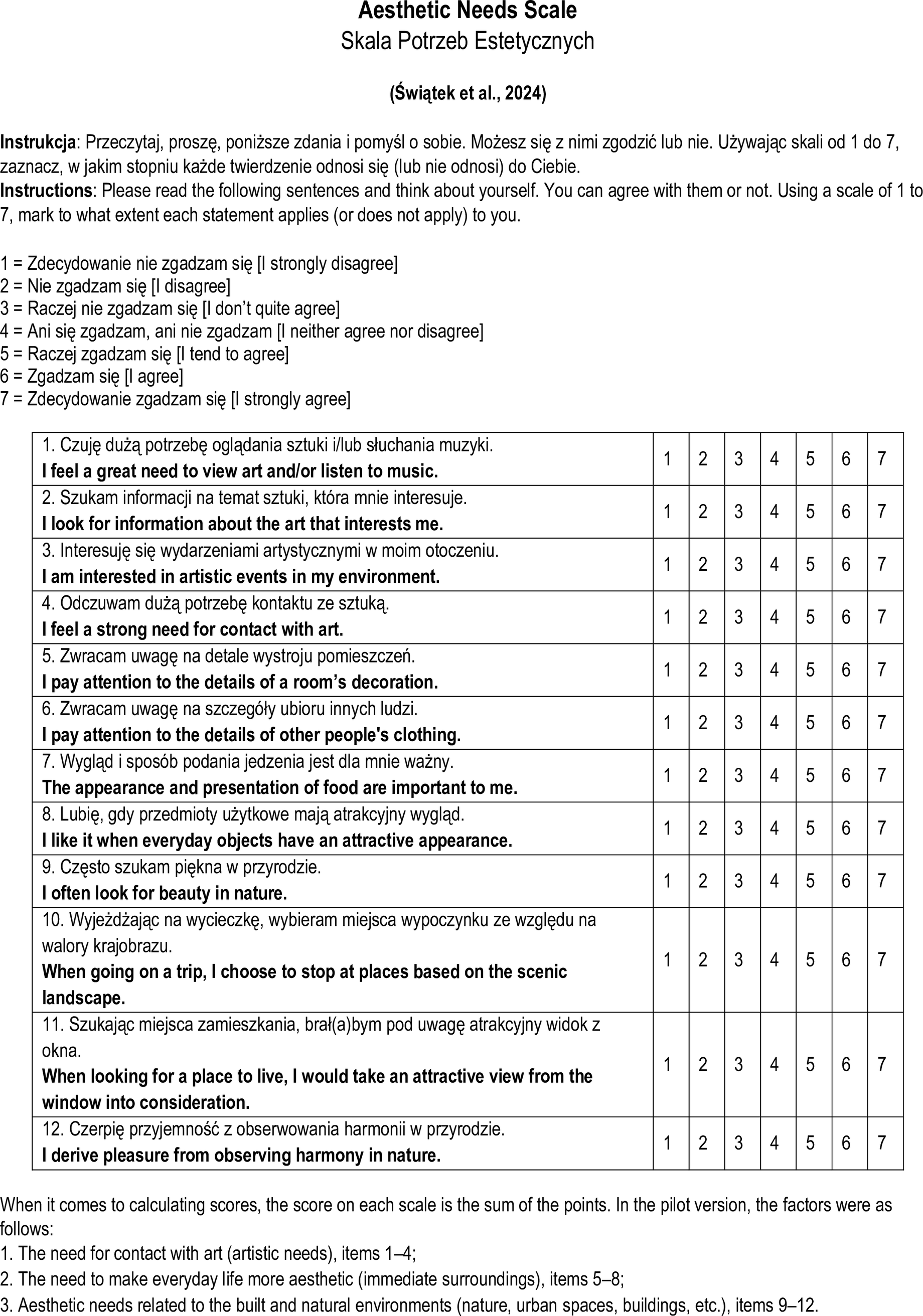

Supplement: S1 Appendix — (TIF) [file pone.0299326.s001.tif]
